# Networks of Care: An Approach to Improving Maternal and Newborn Health

**DOI:** 10.9745/GHSP-D-22-00162

**Published:** 2022-12-21

**Authors:** Katherine Kalaris, Emma Radovich, Andy E. Carmone, Jeffrey Michael Smith, Anne Hyre, Martina Lukong Baye, Clemence Vougmo, Anshu Banerjee, Jerker Liljestrand, Allisyn C. Moran

**Affiliations:** a University of Oxford, Oxford, United Kingdom.; b London School of Hygiene and Tropical Medicine, London, United Kingdom.; c Clinton Health Access Initiative, Boston, MA, USA.; d Bill & Melinda Gates Foundation, Seattle, WA, USA.; e Jhpiego, Baltimore, MD, USA.; f Ministry of Health, Yaoundé, Cameroon.; g Perinatal Network of Yaoundé, Viallaite Cameroun Association, Yaoundé, Cameroon.; h World Health Organization, Geneva, Switzerland.; i Independent consultant, Stockholm, Sweden.

## Abstract

The Networks of Care approach has the potential to harmonize existing strategies and optimize health systems functions for maternal and newborn health, thereby strengthening the quality of care and ultimately improving outcomes.

## Key Messages

- Networks of Care (NOCs) can improve quality of care, continuity of care, and maternal and newborn outcomes. They focus on relational elements that are key to health system functioning and are context specific.
- NOCs focus on creating intentional connections between people and services and strengthening the functional aspects of health systems while incorporating and emphasizing core relational aspects. They can also be used to strengthen referral systems, thus promoting continuity of care.
- With momentum and interest gathering around the NOC approach, it could be an essential tool for meeting the Sustainable Development Goals for maternal and newborn health.

## INTRODUCTION

Despite global progress in reducing preventable maternal and newborn deaths, high rates of maternal and newborn morbidity and mortality persist. Although maternal mortality declined by 44% between 1990 and 2015, this fell significantly short of meeting the 75% target set by the Millennium Development Goals.^1^ An estimated 810 women die each day due to complications of pregnancy and childbirth, with the majority of these deaths taking place in low- and middle-income countries.^2^ The Sustainable Development Goals (SDGs) set a global average maternal mortality target of fewer than 70 deaths per 100,000 live births.^3^ The most recently available global maternal mortality estimate was triple the SDG target, at 211 deaths per 100,000 live births in 2017.^2^ Likewise, newborn mortality remains unacceptably high, with current global estimates of 5,400 stillbirths^4^ and 7,000 newborn deaths every day.^5^ The SDGs aim to reduce newborn mortality to no more than 12 deaths per 1,000 live births,^3^ although most recent estimates put newborn mortality at 17 deaths per 1,000 live births in 2020.^6^

Although access to care has increased, therefore making quality of care a more important determinant of health outcomes in many settings, the increase in access has not been even and remains a serious issue in many settings.^7^ Poor quality of care is now responsible for a larger proportion of deaths than lack of access to care (60% vs. 40%).^8^ Health systems are complex, and care is often suboptimal, fragmented, and poor quality. This is particularly acute for maternal and newborn care, especially when managing time-sensitive, life-threatening complications. It is estimated that 1 million newborn deaths and more than 147,000 maternal deaths could be averted each year by provision of high-quality care.^2^^,^^8^

However, high-quality and equitable care, especially as it relates to maternal and newborn services, cannot exist outside of interconnected systems. Clinicians and facilities working in “systematic coordination” is important for ensuring high-quality care.^9^ Furthermore, a network approach can have important implications for capacity building as it can facilitate innovation and improve communication.^10^ This can be done, for example, by mentoring clinicians within the network.^11^^,^^12^

Although substantial progress has been made in improving maternal and newborn outcomes, achieving the SDGs will require additional transformational and catalytic approaches to accelerate the reduction of maternal and newborn mortality and prevent stillbirths. The Networks of Care (NOCs) approach has the potential to harmonize existing strategies and optimize health systems functions for maternal and newborn health, thereby strengthening quality of care and ultimately improving outcomes. Service delivery networks are not a new approach in practice. For example, primary care networks and integrated delivery systems have been a mechanism to provide care in high-income countries.^13^^,^^14^ However, there is still relatively limited literature and guidance highlighting this approach for maternal and newborn health, particularly in low- and middle-income countries.

The Networks of Care approach has the potential to harmonize existing strategies and optimize health systems functions for maternal and newborn health, thereby strengthening quality of care and ultimately improving outcomes.

## LANDSCAPE REVIEW OF NOCS

The World Health Organization undertook a multiple methods landscape review on NOCs for maternal and newborn health.^15^ The landscape review aimed to develop a working definition of an NOC for maternal and newborn health, assess the scope of the approach in the global landscape, identify recommendations and knowledge gaps for country implementation, and assess the feasibility of the approach at the subnational level. The landscape review consisted of 4 steps: (1) perform a preliminary examination of the published and unpublished literature; (2) develop a working definition of an NOC with the Maternal Health Scoping Review Steering Committee composed of global maternal and newborn experts from 3 United Nations (UN) agencies, researchers, implementors, and policy makers representing all UN world regions; (3) map and synthesize illustrative examples of NOCs for maternal and newborn health; and (4) conduct virtual country-level stakeholder exercises.

### Perform a Preliminary Literature Review

The review of published and unpublished literature on NOCs for maternal and newborn health consisted of searching the PubMed, Scopus, and Web of Science databases and the websites of global health organizations that have worked on this approach (including CARE, Clinton Health Access Initiative, Management Sciences for Health, PATH, and the U.S. Agency for International Development). A search strategy was developed based on the Medical Subject Headings of the U.S. National Library of Medicine and keyword searches for “maternal and newborn health services” and “integrated networks of care” from the year 2000 to the dates on which the searches were run. The database searches were completed June 1–2, 2020, and organization websites were examined during the first 2 weeks of June 2020. Discussions were also held with researchers to understand relevant ongoing research.

The literature search returned 1,901 deduplicated results, but among those, there were no publications specifically labeled as NOCs. Consultations with other researchers at the time of the landscape review confirmed that there was no dedicated literature on NOCs.

### Develop a Working Definition of an NOC for Maternal and Newborn Health

The development of the maternal and newborn NOC definition and supporting key elements was based on earlier work on area-based teamwork for maternal, newborn, and child health^16^ an NOC definition and annotated framework developed through a scoping study with case studies; peer-reviewed and gray literature, and illustrative examples.^9^ Thematic analysis and a template analysis approach were used to synthesize information from the illustrative examples of NOCs (described in the next section) to refine the definition and key elements. The Maternal Health Scoping Review Steering Committee met virtually to craft the iterated definition and key elements of an NOC for maternal and newborn health.

### Map and Synthesize Illustrative Examples of NOCs for Maternal and Newborn Health

The World Health Organization released a call for illustrative examples of NOCs based on a draft definition. Submissions included information on context and time frame for establishing the NOC, how the NOC operationalized elements of the draft definition, and the network’s future plans. A total of 30 examples of NOCs were identified from the submissions and additional searching. The steering committee used deductive thematic analysis to describe the features of the example and how (or not) they operationalized the elements of the NOC definition. Subsequently, the committee used a modified template analysis approach based on the definition and key elements to create a coding template. The coding template was employed to map the extent of the elements appearing in each example according to 3 criteria—clearly present, possibly present, or unknown (not enough information provided). This enabled the researchers to clearly describe the network and how it related to the definition.

The 30 examples represented countries in Africa, Asia, the Americas, and Europe. For 2 of the examples, the researchers were able to assess all elements of the definition based on the information provided and label them as clearly or possibly present. Many of the examples could not be assessed against several elements of the definition due to insufficient reported information; however, this does not necessarily mean that the element was not present in the network. It may also be because some of the relational elements of an NOC can be less concretely measured. In 5 of the 30 examples, only 1 or 2 elements could be assessed based on the available information, raising the question of whether the example could be considered an NOC based on the definition. Two examples are highlighted below; the NOC from Indonesia was identified through the mapping of examples, and the NOC from Cameroon was identified during the virtual country-level stakeholder exercise.

### Conduct Virtual Country-Level Stakeholder Exercises

Virtual country-level stakeholder exercises were undertaken in Cameroon and Nepal to understand how the NOC approach could be implemented at the subnational level. The exercises included a series of meetings and workshops with country maternal and newborn health experts and clinicians, the administration of a questionnaire focused on the relational elements of an NOC, and a preliminary planning activity to identify key steps, resources, and stakeholders necessary to implement an NOC approach for maternal and newborn care at the subnational level. The workshops were facilitated by the World Health Organization country offices and Ministry of Health officials from the Family Health Departments. Workshop participants included maternal and newborn health experts from the Ministries of Health at the national and subnational levels, clinicians, and representatives from professional associations and UN agencies.

During these exercises, participants reflected on why an NOC approach would be appropriate for their context; the key actors involved in governance and leadership of an NOC; how engagement across sectors would occur; and how to strengthen communication, teamwork, trusting relationships, and monitoring. Participants mapped out the next steps for establishing an NOC, which included strategically engaging with and advocating for the approach with the Ministry of Health and stakeholders, establishing norms and policies, reinforcing clinical competencies, ensuring the necessary logistics, and creating a monitoring system.

## DEFINITION OF AN NOC FOR MATERNAL AND NEWBORN HEALTH

The following definition for a maternal and newborn NOC resulted from the process previously described.

*A network of care for maternal and newborn health is a collection of public and/or private health facilities and health workers deliberately interconnected to promote multidisciplinary teamwork and collaborative learning in order to provide comprehensive, equitable, respectful, person-centred care from home/community to primary through to tertiary levels.*

An NOC for maternal and newborn health addresses relational and structural aspects to provide high-quality and continuous care through formal and informal connections and collaboration between and across all levels of care. It engages with transport, communication systems, and other sectors that support care. An NOC is a mechanism for joint accountability between health care workers and individuals and communities for responding to evolving care needs and promoting equitable access to care, including reducing financial barriers.

A functional NOC results in collaborative and coordinated continuity of high-quality, respectful care for maternal and newborn health, from the levels of home and community through to tertiary care, to ultimately optimize linkages for efficient and resilient health systems. NOCs for maternal and newborn health only function within an enabling environment with the structural elements of high-quality, respectful care in place and through supportive health system management structures. The Figure represents the NOC approach in the context of maternal and newborn health; it is centered around the mother-baby dyad and lists the relational and structural elements described below.

**FIGURE.**
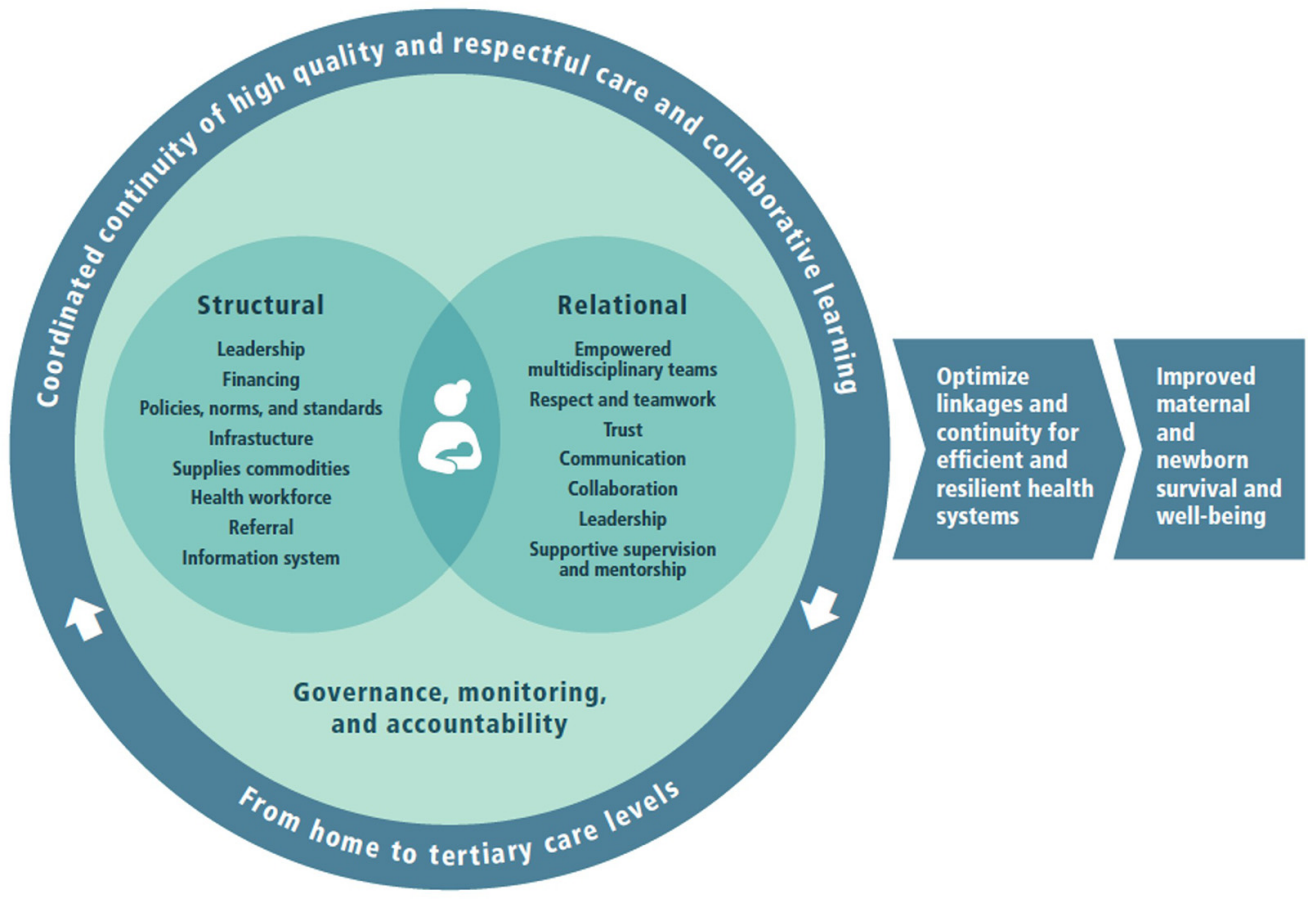
Key Structural and Relational Aspects of Networks of Care for Maternal and Newborn Health

A functional NOC results in collaborative and coordinated continuity of high-quality, respectful care for maternal and newborn health to ultimately optimize linkages for efficient and resilient health systems.

NOCs focus on the following relational aspects—the defining, differentiating aspects of this approach—to strengthen linkages, connections, and collaboration to optimize the functioning of an existing health system.^9^^,^^11^^,^^17^

- Empowered, multidisciplinary, and respectful teamwork based on trust and relationships within and between facilities and levels of care
- Network leadership and stewardship
- Innovative modalities to facilitate communication and collaboration (e.g., WhatsApp groups, telemedicine) among providers, providers and patients, and facilities in the network
- Supportive supervision and mentoring among facilities in the network
- Collaborative learning, sharing, and problem solving
- Monitoring and accountability for achieving network goals

An NOC is not just a referral system but can also strengthen and support an existing referral system through a focus on improving the relational elements, described above, across facility interactions. NOCs do not operate as parallel systems but instead can enhance and improve discrete components of the existing health system to function in a more integrated manner. Existing health system policies and financing can help to facilitate the establishment of an NOC.

## EXAMPLES OF NOCS FOR MATERNAL AND NEWBORN HEALTH

NOCs for maternal and newborn health have been developed in a range of geographical contexts, in urban and rural areas, and at different levels of scale, but they all have the same defining relational elements in common. The Perinatal Network of Yaoundé in Cameroon was identified as an example of an NOC during the virtual country exercises and highlights how an NOC can be initiated by health care workers without additional resources. The Expanding Maternal and Neonatal Survival program in Indonesia was 1 of 2 examples identified in the landscape review that clearly or possibly represented all aspects of the NOC definition.

### Cameroon: Perinatal Network of Yaoundé

In the second largest urban area of Cameroon, Yaoundé, parents of newborns in need of more complex care were left on their own to find a hospital capable of providing the necessary care that would accept the newborn. This erratic search for suitable care—sometimes over long distances, visiting many hospitals without success, and without proper transport—often led to hypothermia and sometimes newborn death.

Maternal and newborn health professionals created a group in WhatsApp, commonly used by health care providers in Cameroon, to coordinate transfer of newborns, forming the Perinatal Networks of Yaoundé.^18^ They formed the network of their own initiative without additional support or funding and members use their own mobile phones and internet connections. Every day, providers share the availability of space and services for newborns in their hospital. Providers with a newborn in need of transfer contact the group to assess the availability of care. If more than 1 facility can offer care, the provider helps the family to select the facility based on their means, as many facilities require out-of-pocket payment. Outcomes of referred newborns are often shared through the group, creating an informal method of information feedback. The group is also used to share best practices and protocols for newborn care.

The network currently has 337 members and is made up of clinicians at 32 hospitals and lower-level facilities, half of which are public facilities, from 8 districts, representatives from the Ministry of Heath, technical and financial partners, and professional associations. For 13 months, from September 2018 to September 2019, 498 neonates were transferred through the network. The main reasons for transfer were need for oxygen, infections, prematurity, and asphyxia. Average response time to requests in the network ranged from 10 to 120 seconds.

Despite having limited resources, the network has solved a major problem of urban emergency referral for newborns. It is a simple approach that has helped improve access to care. This approach has formed the basis of trusting relationships among providers, which are key to its success, and it has incited a change in professional culture. For example, before the establishment of the network, providers were afraid of being criticized for a referral, but the network has helped to reorient the focus on the newborn. It has also reduced stress among providers as now they know how and where to refer cases. The forum facilitates working together, improves capacity of health care workers to provide newborn care, makes communication easier, and promotes clinical discussion of cases. Providers from isolated facilities are linked with other providers and facilities, improving confidence and satisfaction. The network has incited improvements in relationships between providers and newborn caregivers. This has all been accomplished with no dedicated funding. The Table shows how this example met the different elements of the NOC definition.

The Perinatal Network of Yaoundé has solved a major problem of urban emergency referral for newborns and helped improve access to care.

**TABLE.** Indonesia EMAS Program and Cameroon Perinatal Network of Yaoundé Assessment Rated By Elements of the Network of Care for Maternal and Newborn Health Definition

**Network of Care Elements**	**Indonesia**		**Cameroon**	
	**Clearly Present**	**Possibly Present**	**Clearly Present**	**Possibly Present**
Optimizes linkages and the deliberate coordination and governance of service delivery to provide comprehensive MNH care from community to referral levels	X		X	
Addresses relational and functional aspects of provision of high-quality, continuous care	X		X	
Includes formal and informal connections, collaboration, and reporting between and across levels, including primary and specialty care	X		X	
Fosters respectful teamwork and communication between all health care providers at different levels	X		X	
Engages with transport, communication (mobile phone or radio), and other sectors that support care	X			X
Incorporates mechanisms for joint accountability by health workers with individuals and communities and for responding to evolving care needs		X		X
Promotes equitable access to care including reducing financial barriers		X		X
Measures and monitors health processes and outcomes for women and newborns	X			X

Abbreviations: EMAS, Expanding Maternal and Neonatal Survival; MNH, maternal and newborn health.

### Indonesia: Expanding Maternal and Neonatal Survival

In Indonesia, to improve maternal and newborn survival, district-level NOCs were formed across 30 districts in 6 provinces, including more than 400 public and private referral hospitals and health centers, where over half of all maternal deaths in the country occurred. The goals of the networks were to improve the quality of hospital and health center emergency obstetric care, increase referral system efficiency and effectiveness, and increase accountability. These networks were implemented by a consortium of partners (Jhpiego; Lembaga Kesehatan Budi Kemuliaan maternity hospital; Muhammadiyah, an Islamic social and education organization; Research Triangle Institute International; and Save the Children) supporting the government at national, provincial, and district levels and collaborating with civil society, hospital associations, professional organizations, and private sector agencies. The Table shows how this example met the different elements of the NOC definition.

Existing government policies encouraged facility-based births and the provision of high-quality maternal and newborn services. Universal health coverage for maternal and newborn services was being rolled out nationally to reduce financial barriers. The networks were supported by memorandums of understanding with agreed-upon referral pathways and roles and responsibilities of health facilities around care, referrals, mentoring, and service delivery payment. An automated communication system supported the referral networks. Multisectoral district-level working groups met quarterly with network members to review health facility data, identify and propose solutions to problems, and develop action plans. Peer-to-peer mentoring was implemented, with high-performing facilities and districts mentoring other facilities to build capacity for improving clinical governance, quality, the referral system, and accountability. This fostered long-term relationships between facilities, communities, and districts and promoted accountability, communication, and continual learning. Other facility-based activities included maternal and newborn death reviews; emergency drills to improve clinical skills, teamwork, and communication; and improved use of data for decision making, which facilitated joint problem solving. This combination of efforts led to improved collaboration and trust among providers—both within individual facilities and across facilities in the network. The network extended to the community level through volunteer village motivators to support maternal and newborn survival initiatives and civic forums to provide feedback and monitoring and to promote use of services.^18^

Through collaboration and advocacy with the districts, the network expanded to additional facilities in most of the intervention districts and expanded to 35 additional districts/cities with local funding. The network has continued to function in at least 15 districts following the end of external funding.

The network improved the capacity of providers and facilities in the network and clinical outcomes. A quasi-experimental study found a mean difference in quality scores between network hospitals and comparison sites for facility-based labor and childbirth care. The Expanding Maternal and Neonatal Survival intervention hospitals had better performance in labor monitoring (14 points higher), newborn resuscitation readiness (38 points higher), and infection prevention practices (33 points higher).^20^ An assessment of longitudinal monitoring data found improvements in the stabilization practices prereferral for preeclampsia/eclampsia (24% to 61%) and the treatment of newborns with suspected severe infection (30% to 54%).^21^ The effectiveness of referral improved when pregnant women were referred through the automated electronic referral exchange system due to higher levels of effective communication and advanced notification of referral.^21^ However, there was not a significant difference in referral efficiency as the lag time between the decision to refer and departure was similar in facilities with and without the automated electronic referral exchange system.^21^ Accountability was increased through (1) working with local government by setting up province- and district-level technical working groups focused on shared governance, problem solving, collaboration, and taking action and (2) civic engagement by holding civic forums to empower civil society in decision making.^19^ An evaluation of the network showed positive results, with the case fatality rate among women admitted with any maternal complications to hospitals in the network decreasing by 50% (from 5.4 to 2.6 deaths per 1000 cases of admitted obstetric cases) and very early newborn mortality decreasing by 21% during the period of the program.^22^

The Indonesia NOC improved the capacity of providers and facilities in the network and clinical outcomes.

## CONCLUSION

The NOC approach can be used to improve quality of care, continuity of care, and maternal and newborn outcomes. NOCs focus on relational elements that are key to health system functioning and are context specific. An NOC is not a parallel system to the existing public and private health systems but an optimization of the existing system that can facilitate continuity of care throughout pregnancy, childbirth, and the postpartum period and from the community to tertiary levels. NOCs focus on creating intentional connections between people and services and strengthening the functional aspects of health systems while incorporating and emphasizing core relational aspects. They can also be used to strengthen referral systems by promoting continuity of care and improving the capacity of providers.

The NOC approach can target a specific clinical issue or health system problem or can take on broader primary health care implementation, such as care networks that have been developed as the basis of the Brazilian health care system,^23^ primary care networks in the United Kingdom,^13^ and integrated service delivery systems in the United States and Europe.^14^ In Brazil, care networks have emerged as a solution for integrating health services to improve resilience and respond more effectively to the complex health needs of the population. Health facilities are horizontally linked together by a single mission, common objectives, and cooperative and interdependent action to enable continuous and comprehensive care to a population in a defined geographic area coordinated by primary health care. Care is centered around the individual, family, and community. Maternal and perinatal networks are integrated into these primary care networks and include maternity waiting homes, community birth centers, maternity hospitals, referral hospitals, emergency services, and laboratory and diagnostic services. The health system in Brazil exemplifies a way that networks can be scaled across a health system. Other countries, such as Ghana, are also embarking on a national NOC approach for primary health care. However, due to their context-specific nature, NOCs will likely be scaled in different ways between countries.

An NOC is a particularly appropriate strategy for maternal and newborn care, which can be complicated and time-sensitive, because it focuses on the underlying relational elements, such as teamwork, trust, collaboration, and communication, that are essential to providing high-quality, respectful care. It is also an approach that supports continuity of care, which is essential for maternal and newborn care and can bridge what are often siloed and fragmented services.

### Future Considerations

Despite many examples in practice (the landscape review identified 30 examples), NOCs are still relatively underresearched. With documented evidence of their success in improving health system functioning and clinical outcomes, particularly for maternal and newborn care, and a growing interest in implementation, there is an important opportunity to further understand the success of these networks to provide guidance for future implementation. We must also identify ways to measure the success and effectiveness of NOCs and understand their role in coordinating and optimizing the impact and sustainability of more targeted interventions in maternal and newborn health. Next steps may include the following.

- Implement, document, and evaluate Networks of Care on maternal and newborn outcomes
- Encourage opportunities for sharing and learning from ongoing experiences
- Provide guidance for NOC country implementation, monitoring, and learning

With momentum and interest gathering around the NOC approach, it could be essential to meeting the SDGs for maternal and newborn health.

## Notes

**Peer Reviewed**
